# Enhancement of T cell immunity to osteosarcoma through modulation of PD-1/PD-L1 interactions

**DOI:** 10.1186/2051-1426-1-S1-P162

**Published:** 2013-11-07

**Authors:** Danielle M Lussier, Lizbeth M Nieves, Megan S McAfee, Trung P Hyunh, Susan A Holechek, Pooja Hingorani, Joseph N Blattman

**Affiliations:** 1 School of Life Sciences, Arizona State University, Tempe, AZ, USA; 2 Center for Infectious Diseases and Vaccinology, Arizona State University, Tempe, AZ, USA; 3 Center for Cancer and Blood Disorders, Phoenix Children's Hospital, Phoenix, AZ, USA

## 

Osteosarcomas remain one of the most commonly occurring bone cancers in adolescents, accounting for approximately 3% of all diagnosed childhood malignancies. Approximately 30% of osteosarcoma patients develop pulmonary metastatic disease, which is associated with poor prognosis, as 5-year survival rates for these patients are less than 20%. Cytotoxic T-lymphocytes (CTL) likely play an important role in the control of developing osteosarcomas as polymorphisms in cytotoxic T lymphocyte antigen 4 (CTLA-4), an inhibitory tumor necrosis factor receptor (TNFR) family member, result in increased CTLA-4 protein expression levels, and are associated with higher risk of developing osteosarcoma. While CTLA-4 is involved in limiting the generation of CTL responses, other inhibitory TNFR family members limit the function of CTL after tumor metastases and during disease progression. In particular, ligation of programmed death receptor-1 (PD-1), expressed on tumor-specific CTL, by PD-1 ligand (PD-L1) on tumors, results in potent inhibition of CTL proliferation, cytokine production, and cytotoxicity, leading to tumor progression. Using a mouse model of metastatic osteosarcoma, we have shown that PD-1 is expressed on osteosarcoma tumor infiltrating CTL, and that osteosarcoma cells express PD-L1. Human metastatic osteosarcomas also exhibit increased expression of PD-L1 compared to pre-metastatic tumors, with PD-1 expression on metastatic tumor infiltrating lymphocytes. Furthermore, we have shown that removal of PD-1/PD-L1 interactions in the mouse metastatic osteosarcoma models dramatically improves survival outcomes. These studies of the role of PD-1 in inhibition of CTL-mediated eradication in metastatic osteosarcoma in mouse models and in primary human metastatic osteosarcoma provide the necessary pre-clinical data required for subsequent clinical testing of therapeutic approaches to block PD-1/PD-L1 interactions for treatment of metastatic osteosarcoma patients.

